# Possible Role of Autophagy in the Treatment of Pancreatic Cancer with Histone Deacetylase Inhibitors

**DOI:** 10.3390/cancers2042026

**Published:** 2010-11-29

**Authors:** Hidemi Rikiishi

**Affiliations:** Department of Microbiology and Immunology, Tohoku University Graduate School of Dentistry, 4-1 Seiryo-machi, Aoba-ku, Sendai 980-8575, Japan; E-Mail: riki@dent.tohoku.ac.jp; Fax: +81-22-717-8309

**Keywords:** pancreatic cancer, autophagy, apoptosis, epigenetics, histone deacetylase, ER stress

## Abstract

Pancreatic cancer is a lethal disease and notoriously difficult to treat. Only a small proportion is curative by surgical resection, whilst standard chemotherapy for patients with advanced disease has only a modest effect with substantial toxicity. Clearly there is a need for the continual development of novel therapeutic agents to improve the current situation. Currently, there is a bulk of data indicating the important function of autophagy in cancer. While genetic evidence indicates that autophagy functions as a tumor suppressor, it is also apparent that autophagy can promote the survival of established tumors under stress conditions and in response to chemotherapy. This review provides a spectrum of potential pharmacological agents and autophagic approaches to enhance cell killing in pancreatic cancer.

## 1. Introduction

Pancreatic cancer lacks specific symptoms and early detection tools and thus, has the worst mortality rate and the lowest overall survival of all cancers. In particular, pancreatic ductal adenocarcinoma is an extremely aggressive and devastating neoplasm, which often invades and destroys surrounding stromal components, including lymphatic, vascular, and perineural systems, ultimately metastasizing to distant organs. Another reason for the poor prognosis of pancreatic cancer is the insensitivity to most therapies, such as chemotherapy, radiotherapy and immunotherapy. Thus, novel strategies to treat this deadly disease are urgently needed.

Chemotherapy is still the only option in metastatic pancreatic cancer treatment. In particular, gemcitabine has represented the standard chemotherapy for all stages of pancreatic adenocarcinoma in the last decade; however, neither gemcitabine alone nor gemcitabine-based combinational chemotherapy achieves a favorable outcome in advanced cases. New adjuvant therapy targeting specific markers in pancreatic cancer using the molecular approach may represent a promising strategy in the diagnosis and treatment of pancreatic cancer. Indeed, these molecular approaches have rapidly developed in recent years, and include anti-sense oligonucleotides, RNA interference, gene restoration, suicide gene therapy, small molecule inhibitors, oncolytic viral therapy, and antibody therapy. However, many of those approaches have yet to be tested in clinical applications, and most of the treatments need to be combined with standard chemotherapy or radiotherapy for maximum benefits [1].

## 2. New Molecular Approaches to Pancreatic Cancer Treatment

Gemcitabine (2’-2’-difluorodeoxycytidine) is the most active chemotherapy agent used for the treatment of pancreatic cancer. It is an analog of deoxycytidine that is incorporated into double-stranded DNA during the S (synthesis) phase, resulting in inhibition of DNA synthesis, arrest of cell cycle progression, and induction of apoptosis. In general, resistance to chemotherapy, whether intrinsic or acquired, is believed to be a major cause of treatment failure in pancreatic cancer [2]. It is certainly considered that resistance to gemcitabine treatment is mainly attributed to an altered apoptotic threshold in pancreatic cancer cells [3]. Gemcitabine is also incorporated into RNA, depending on the cell line, at similar levels as in DNA, resulting in RNA synthesis inhibition in human cell lines. Sensitivity to gemcitabine seems to be related to differences in RNA incorporation. However, collateral sensitivity to gemcitabine in doxorubicin-resistant cells was related to an increased incorporation into DNA but not to RNA incorporation. Attempts to better understand the molecular basis for these characteristics of pancreatic cancer have focused on studying the gene and protein expression profiles of both resected tumors and pancreatic cancer cell lines. Accordingly, tumor suppressor genes related to anti-oxidant activity, apoptosis, the cell cycle, signal transduction and intracellular adhesion, are often mutated or silenced by epigenetic modifiers, such as histone deacetylases (HDACs) and DNA methyltransferases (DNMTs). Recent studies have shown that clinically relevant HDAC inhibitors, such as suberoylanilide hydroxamic acid (SAHA), can restore sensitivity to gemcitabine and other agents [4]. Thus, it may be possible to use HDAC inhibitors to restore drug sensitivity to pancreatic cancers. Eventually, a strategy for sensitizer (lowering the apoptotic threshold)/inducer (activation of the apoptotic machinery), could be a potential approach for a rational molecular-based tumor therapy for pancreatic cancer.

In addition, a recent study showed that blocking sphingosine kinase-1 (SphK1) activity using small interfering RNA (siRNA) can sensitize pancreatic cancer cells to gemcitabine treatment, indicating that development of combinational therapy of siRNA with gemcitabine may represent a promising approach in pancreatic cancer treatment [5]. siRNA-mediated silencing of anti-apoptotic Bcl-2 enhances chemotherapy sensitivity in human pancreatic cancer cells *in vitro*. Moreover, replacement of *p53* in pancreatic cancer cells previously treated with gemcitabine increased cytotoxicity both *in vitro* and *in vivo* [6]. *HSV-TK* (herpes simplex virus-thymidine kinase) gene delivery followed by gancyclovir was found to be effective in inhibiting the tumor growth and metastasis of pancreatic cancer [7]. Tissue-specific promoters are preferred in suicide gene therapy in order to achieve maximal efficacy and minimal toxicity. Erlotinib, a small molecule tyrosine kinase inhibitor, targets the intracellular domain of the epidermal growth factor receptor (EGFR), and has been shown to improve survival when used in combination with gemcitabine to treat metastatic pancreatic cancers [8]. In addition, a patient with stage IV pancreatic cancer showed a response to chemotherapy with the addition of bevacizumab, a recombinant humanized monoclonal antibody targeting vascular endothelial growth factor (VEGF), while it was initially unresponsive to gemcitabine, 5-fluorouracil, irinotecan, and cisplatin treatment [9]. These therapeutic agents will provide a new perspective on pancreatic cancer treatment using molecular approaches.

## 3. Genetic Background of Pancreatic Cancer Cells

Cancer cells express the successive accumulation of gene mutations (oncogenes, tumor-suppressor genes, and stability genes), which increase aggressiveness and confer resistance to conventional chemotherapy and radiotherapy [10]. In particular, pancreatic cancer cells seem to differ from cervical, prostate, colorectal, and small bowel cancer cells in their expression of Bcl-xL, Bcl-2, cyclin D1, and TRAIL decoy receptor 2 (DcR2) [11]. Moreover, the *p53* mutation occurs in 50–75% of infiltrating pancreatic adenocarcinomas, being rare in chronic pancreatitis [12]. p53 plays a central role in modulating cellular responses to cytotoxic stresses by contributing to both cell-cycle arrest and apoptosis. Mutations in cyclin-dependent kinase inhibitor *p16* and *Smad4*, a downstream target of transforming growth factor β, also exhibit high mutation frequencies in pancreatic tumors [13]. Activation of the *K-Ras* oncogene has been implicated in more than 90% of pancreatic carcinogenesis, and *K-Ras* mutation represents one of the earliest genetic alterations in pancreatic cancer development [14]. Chemotherapeutic resistance is often associated with mutations in codon 12 of the *K-Ras* gene [15]. Oncogenic K-Ras promotes pancreatic tumorigenesis through the activation of multiple downstream pathways, including PI3K/Akt, ERK, Bad, and NF-κB [16]. Moreover, overexpression of p21^WAF1/CP1^ (wild type p53 activated fragment-1/poly(c)-binding protein 1) has been reported to be an early event in the development of pancreatic intraepithelial neoplasia [17]. Resistance to apoptosis is also a hallmark of pancreatic cancers, and therefore lowering the apoptotic threshold is a therapeutic goal [18]. NF-κB is constitutively active in pancreatic cancers, which protects the cells from apoptosis and, in some cases, stimulates their growth. TNF-α-induced apoptosis is inhibited by the concomitant overexpression of NF-κB signaling induction, in which free active NF-κB migrates into the nucleus, inducing the transcription of different anti-apoptotic genes, such as *Cox-2*, *IAPs*, *XIAP*, *Survivin*, *Bcl-xL*, *Bcl-2*, and *FLIP* (Flice-like inhibitory protein) [19].

## 4. Epigenetic Alterations in Pancreatic Cancer

Such genetic backgrounds originate from missense mutations at residues that are essential for activity, from mutations that result in a truncated protein, from deletions or insertions of various sizes, or from epigenetic silencing. Epigenetic alterations are defined as heritable changes in gene expression that are not accompanied by changes in DNA sequence. The past several years have provided an explosive increase in our knowledge of epigenetic features in human cancers. Along with genetic events, tumor-associated epigenetic alterations are important determinants in the initiation and progression of pancreatic cancer [20]. Indeed, pancreatic cancers harbor numerous epigenetic alterations, these alterations can be observed in neoplastic precursor lesions, and their prevalence increases as lesions become more advanced [21]. Specific events driving changes that lead to cancer development and progression are interconnected complex molecular modifications, including DNA methylation, histone acetylation, phosphorylation, ubiquitylation and ADP ribosylation.

### 4.1. Aberrant DNA Methylation

Epigenetic alterations in transformed cells involve changes in DNA methylation, including global hypomethylation and locus-specific hypermethylation. DNA methylation is an enzyme-driven chemical change to the DNA sequence that most commonly occurs at CpG dinucleotides in mammals. The global methylated cytosine content is often reduced in cancer, including pancreatic cancers. One consequence of genome-wide hypomethylation may be genomic instability, a characteristic of most pancreatic cancers. By contrast, hypermethylation of *p16^INK4A^* in a subset of pancreatic cancers was one of the early reports of aberrant methylation in pancreatic cancers. The *p16^INK4A^* is inactivated in approximately 95% of pancreatic adenocarcinomas, approximately 15% of which were attributable to aberrant promoter methylation. The *p16^INK4A^* gene is aberrantly methylated in 27% of pancreatic cancer cell lines [22]. The reversal of methylation by a DNMT inhibitor, 5-aza-2’-deoxycytidine (5-Aza-dC), results in increased mRNA expression of epigenetically-relevant genes. It has been shown that 5-Aza-dC inhibits the growth of pancreatic cancer cell lines. In addition, pretreatment with 5-Aza-dC increased the sensitivity of pancreatic cancer cells to other chemotherapy agents, including TNF-α, cisplatin, and gemcitabine [23]. DNMT inhibition by zebularine showed a promising anti-cancerous effect in pancreatic cancer cells *in vitro* and *in vivo*, leading to apoptosis induction, growth suppression and phenotypic stabilization. This effect could be augmented by co-incubation with the HDAC inhibitor SAHA *in vitro*, but was not observed in the xenograft model [24].

### 4.2. Histone Modifications

Histone acetylation/deacetylation alters the status of open chromatin domains and thus affects gene transcription. This process is modulated by histone acetyltransferases (HATs) and HDACs. Acetylation correlates with the remodeling of nucleosomes, resulting in relaxation of the chromatin structure which facilitates the accessibility of a variety of factors to DNA causing transcriptional activation. In contrast, deacetylation of the histone tails induces transcriptional repression through chromatin condensation. Locus-specific changes in histone acetylation have been linked to the altered expression of several critical genes in pancreatic cancer, whereas widespread changes in gene expression after the treatment of cell lines with HDAC inhibitors suggest that histone modifications may play a much broader role in regulating gene expression in pancreatic cancer [25]. HDAC inhibitors seem to be specifically selective against tumor cells and show very low toxicity [26]. For example, HDAC inhibitors trichostatin A (TSA) [27,28], SAHA [4] and FK228 [29] have exerted growth-inhibitory and pro-apoptotic effects on pancreatic cancer cell models. Combined treatment with TSA and gemcitabine synergistically inhibited the growth of four pancreatic adenocarcinoma cell lines and induced apoptosis. This effect was associated with the induction of reactive oxygen species (ROS) by gemcitabine, and increased the expression of the pro-apoptotic *Bim* gene by both TSA and gemcitabine. *In vivo* studies on xenografts of pancreatic adenocarcinoma cells in nude mice showed that the association of TSA and gemcitabine reduced the tumor mass by 50% and did not cause any apparent toxicity, while treatments with TSA or gemcitabine alone were ineffective [27].

## 5. HDAC Expression in Pancreatic Cancer

Histone acetylation of the ε-amino site of lysines located at the N-terminal tail of histones by HAT plays a key role in the regulation of transcription by modulating the structure of chromatin, and is precisely regulated by the balance between the activities of HAT and HDAC. HDACs deacetylate and lead to repressive chromatin formation (heterochromatin) and the suppression of gene expression [30]. In addition to the condensation of chromatin, non-histone proteins have been identified as acetylation targets, and reversible lysine acetylation in these proteins plays an important role(s) in the regulation of mRNA stability, protein localization and degradation, and protein-protein and protein-DNA interactions. Many of these proteins are transcription factors, such as p53, C/EBPβ, NF-κB and STATs. A common finding in several cancer cells is high HDAC expression levels and, consequently, a low level of histone acetylation when compared with normal tissues (Table 1). The majority of reports has focused on class I HDACs and suggests the clinical manifestations of aberrant HDAC expression. For example, in cancers of the gastrointestinal system, high HDAC1, HDAC2, and HDAC3 expression correlated with a poor clinical outcome [31], whereas HDAC5 mRNA transcript was shown to be down-regulated in colon cancer [32]. In neuroblastoma, among all HDAC family members investigated, only HDAC8 was associated with advanced-stage disease and poor prognosis [33]. Moreover, SIRT8 was shown to be overexpressed in thyroid carcinoma and cell lines but not in adenomas [34], whereas the *SIRT2* gene was shown to be down-regulated in human gliomas [35]. The expression of individual HDAC family members therefore seems to be tumor specific.

In human pancreatic cancers (Table 2), 56% of pancreatic cancers show positive immunehistochemical staining for HDAC1, and the co-expression of HDAC1 and hypoxia-inducible factor-1α (HIF-1α) markedly correlates with poor prognosis [36]. HDAC2, which is highly expressed, controls resistance to TRAIL. Using tissue microarrays, the overexpression of HDAC2 was detected, especially in moderately differentiated (G2) and undifferentiated (G3) pancreatic cancers [37]. At the molecular level, HDAC2 inhibition opens the locus of the epigenetically silenced *NOXA* gene, a BH3-only protein and apical initiator of apoptosis [37]. In addition, nine of the 11 pancreatic adenocarcinomas (approximately 81%) showed a significant increase of HDAC7 mRNA levels [38]. It has been demonstrated that HDAC7 may regulate the initiation of apoptosis [39]. A recent study has reported that the SAHA, the first HDAC inhibitor approved for clinical use in the treatment of the cancer cutaneous T-cell lymphoma [40], selectively suppresses the expression of HDAC7 [41]. Down-regulation of HDAC7 by SAHA is more pronounced in transformed cells sensitive to inhibitor-induced cell death than in normal cells or cancer cells resistant to induced cell death. These data suggest a role for HDAC isoform overexpression in pancreatic cancers; however, a more detailed investigation of HDAC expression in pancreatic cancers is necessary, especially in larger cohorts and in correlation with clinical and prognostic parameters.

**Table 1:** HDAC expression in cancer.

Tumor type	HDAC isoform	Expression	Ref.
Breast cancer	HDAC4, HDAC6	Increase	[66]
Colon cancer	HDAC1, HDAC2, HDAC3, HDAC8	Increase	[67]
Colorectal cancer	HDAC1, HDAC2, HDAC3, SIRT1	Increase	[68]
Gastric cancer	HDAC1, HDAC2, HDAC3	Increase	[31]
Glioma	SIRT2	Decrease	[35]
Lung cancer	HDAC1, HDAC3 HDAC5, HDAC10	Increase Decrease	[69]
Neuroblastoma	HDAC8	Increase	[33]
Oral cancer	HDAC2, HDAC6	Increase	[70]
Ovarian cancer	HDAC3	Increase	[71]
Prostate cancer	HDAC1, HDAC2, HDAC3	Increase	[72]
Thyroid carcinoma	SIRT8	Increase	[34]

**Table 2:** HDAC expression in pancreatic cancer.

HDAC isoform	Function	Ref.
HDAC1	Poor prognosis Chemotherapy resistance Resistance to cell cycle arrest, growth inhibition, and apoptosis Autophagy resistance Repression of promoters of tumor suppressor genes Induction of proliferation and dedifferentiation	[36]
HDAC2	TRAIL resistance Etoposide resistance *NOXA* gene silencing Resistance to differentiation, apoptosis and p53 independent p21 expression	[37]
HDAC7	Apoptosis initiation Mitochondrial localization Malignant progression Overexpression of PDGF-B Control angiogenesis through regulation of angiogenic genes	[38,39]

## 6. Induction of Autophagy by HDAC Inhibitors

The ubiquitin–proteasome system and autophagy are two major intracellular pathways for protein degradation. The ubiquitin–proteasome system degrades short-lived proteins whose functions are usually critical to the control of cell proliferation and cell death. Autophagy is an evolutionarily conserved catabolic process where a cell self-digests its cytoplasmic contents. Autophagy is a key mechanism for long-lived protein degradation and organelle turnover, and serves as a critical adaptive response that recycles energy and nutrients during starvation or stress [42]. Autophagy is tightly regulated by a limited number of highly conserved genes called autophagy regulators (Atgs); however, whether *Atg* genes work through their expected mechanisms of autophagy regulation and/or through as-yet-undefined functions in the development of cancer remains to be further clarified. Autophagy is activated in response to multiple stresses during cancer progression, such as nutrient starvation, unfolded protein response, endoplasmic reticulum (ER) stress, and hypoxia (Table 3); it is also observed upon treatment of cancers with a wide spectrum of cytotoxic and targeted chemotherapeutic agents [43]. Recent findings indicate that suppression of the ubiquitin–proteasome system by proteasome inhibitors induces autophagy through multiple pathways, including activation of HDAC6, which deacetylates α-tubulin in the cytoplasm [44]. It is thought that the deacetylation of α-tubulin by HDAC6 is necessary for transport of the aggresome by microtubules to the lysosome for degradation. The role of autophagy as a survival mechanism in response to these diverse stressors has been well established. Moreover, it has become increasingly clear that a basal level of autophagy serves housekeeping functions vital for maintaining cellular homeostasis; specifically, the failure to clear protein aggregates or damaged organelles via autophagy has been implicated in multiple pathological conditions, including cancer [45].

**Table 3:** Autophagy modulators and their mode of action.

Mode of action	Modulator	Effect on autophagy	Ref.
mTORC1 inhibition	Rapamycin, CCI-779, Rottlein, Curcumin	Activator	[73]
Akt inhibition	Perifosine, Curcumin, Resveratrol	Activator	[74]
GSK3 βP inhibition	Lithium	Activator	[75]
Inositol and IP_3_ reduction	Sodium valproate	Activator	[76]
Ca^2^^+^ level reduction	Verapamil	Activator	[77]
Calpain inhibition	Calpastatin	Activator	[78]
cAMP level reduction	Clonidine	Activator	[77]
HDAC inhibition	SAHA, Butyrate, Sodium valproate	Activator	[47]
Tyrosine kinase inhibition	Imatinib	Activator	[79]
PI3 kinase inhibition	LY294002, Wortmannin, 3-Methyladenine	Inhibitor	[80]
p38 MAP kinase inhibition	SB202190	Inhibitor	[81]
Bcl-2, Bcl-xL inhibition	Arsenic trioxide, ABT737	Activator	[82]
Lysosomotropic drug	Chloroquine	Inhibitor	[83]
HSP70 inhibition	2-Phenylethynesulfonamide	Activator	[84]
Tubulin inhibition	Vincristine, Paclitaxel	Inhibitor	[85]
Cytochrome c release	Resveratrol	Activator	[74]
Atg5 function	Phenethyl isothiocyanate	Activator	[86]
Dopamine antagonist	Fluspirilene	Activator	[87]
ER antagonist	Tamoxifen	Activator	[88]
DNA damage	Radiation	Activator	[89]

To investigate if a class I HDAC isotype is involved in autophagy, a specific class I HDAC inhibitor and an siRNA of HDAC1 were used to treat HeLa cells. Both inhibition and genetic knock-down of HDAC1 in the cells significantly induced autophagic vacuole formation and lysosome function [46]. Two distinct HDAC inhibitors, butyrate and SAHA, induced caspase-3 activation and cell death in multiple human cancer cell lines; however, *Apaf-1* knockout, overexpression of Bcl-xL, and pharmacological inhibition of caspase activity did not prevent SAHA and butyrate-induced cell death. The cells undergoing such caspase-independent death had unambiguous morphological features of autophagic cell death. Induction of autophagic cell death by HDAC inhibitors has clear clinical implications in treating cancers with apoptotic defects [47]. HDAC inhibitor (SAHA) induced autophagy in cancer cells through inhibition of Akt/mTOR pathway and induction of ER stress response. Inhibition of autophagy reduced SAHA-induced cytotoxicity, indicating that SAHA elicited autophagic cell death. SAHA is an attractive candidate for the treatment of pancreatic cancer and pharmacological targeting of autophagy provides promise for the management of cancer therapy. Autophagy is negatively regulated by the class I PI3K signaling pathway. SAHA diminished mTOR expression and mediated caspase-independent cytotoxicity in endometrial sarcoma cells via autophagic mechanisms [48]. Furthermore, HDAC inhibitor FK228 (depsipeptide) inhibited proliferation and induced apoptosis in the malignant rhabdoid tumors cell lines tested. Preincubation with the pancaspase inhibitor zVAD-fmk did not completely rescue FK228-induced cell death, although it did inhibit apoptosis. Disrupting autophagy with chloroquine treatment enhanced FK228-induced cell death [49]. HDAC inhibitor TSA induced AIF release from the mitochondria in human pancreatic adenocarcinoma cell lines [50], but no study has suggested that AIF translocation into the nucleus mediates autophagy. Autophagy rather than the apoptosis-inducing activity for both H40 and SAHA was observed in prostate cancer PC-3M cells [51]. HDAC inhibitor-induced expression of p21^WAF1/CP1^, a modulator of apoptosis, was evident in PC-3M and HL-60 cell lines; however, it is not known whether the inability to induce apoptosis by these drugs in PC-3M cells can be attributed to their induction of p21^WAF1/CP1^ expression (Table 4).

**Table 4:** Effects of HDAC inhibitors in pancreatic cancer cells.

HDAC inhibitor	Pancreatic cell line	Effects of HDAC inhibitor	Ref.
Benzyl isothiocyanate	BxPC-3, Capan-2	Growth suppression	[90]
Valproic acid	MiaPaCa2, Panc1	TRAIL sensitivity	[91]
Valproic acid	MiaPaCa2, Panc1, BxPc3	Etoposide sensitivity	[37]
SAHA	Panc1, BxPC-3	Gemcitabine sensitivity	[92]
Depsipeptide	Panc1	Heterochromatin-associated protein 1	[93]
Trichostatin A	MiaPaca2, PaCa3, Panc1	Gemcitabine sensitivity	[27]
4-Phenylbutyrate	Panc1, T4M-4, BxPc3	Gemcitabine sensitivity	[94]
Trichostatin A	MiaPaca2, PaCa3, Panc1	Gemcitabine, 5-fluorouracil sensitivity	[95]
FR901228	Capan-1, BxPC-3, Panc-1, MIAPaCa-2	Apoptosis induction	[29]

## 7. Autophagy in Pancreatic Cancer Cells

Autophagy is increased in pancreatic cancer cells in resected tumors and correlates with poor patient outcome [52]. Hypoxia in pancreatic cancer has been reported to increase its malignant potential. Autophagy is thought to be a response to factors in the cancer microenvironment, such as hypoxia and poor nutrient supply. Autophagic capacity is elevated in the earliest premalignant lesions and remains high, although it varies throughout premalignant progression, but drops with the appearance of adenocarcinoma [53]. Both murine and human pancreatic tumor cell lines showed clear evidence of enhanced autophagy following treatment with chemotherapeutic agents. In addition, most pancreatic cancers with mutations in apoptotic pathways illuminate the importance of autophagic cell death [54]. In contrast to apoptosis, cell death associated with autophagy is caspase-independent and does not involve nuclear fragmentation.

### 7.1. Triptolide-Induced Autophagy

A diterpene triepoxide, triptolide, was extracted from a Chinese herb that inhibited the proliferation of cancer cells *in vitro* and reduced the growth and metastases of tumors *in vivo*. Knock-down of caspase-3 using the siRNA pool in pancreatic cancer cell lines S2-013 and S2-VP10 cells did not affect cell viability after triptolide treatment [55], suggesting the involvement of a non-apoptotic and caspase-independent cell death pathway in these cells. The induction of autophagy was confirmed by the following responses to triptolide: the increase in the LC3-II form is both time- and dose-dependent; triptolide-treated S2-013 tumors also show an increase in LC3-II *in vivo*; the increase in acridine orange staining in response to triptolide can be reversed by the addition of an autophagy-specific inhibitor, 3-methyladenine. Knock-down of *atg5* or *beclin1* genes, essential in autophagy, did not prevent triptolide-mediated cell death in pancreatic cancer cell lines but instead triggered apoptosis, whereas dual silencing of *beclin1* and *caspase-3* rescued triptolide-mediated cell death. Evaluating the effect of a stimulator on these regulators of the autophagic and apoptotic pathway will aid in explaining why different pancreatic cancer cells have a differential response to stimulator. In pancreatic adenocarcinoma, *K-ras*, *p53*, *p16*, and *DPC4* genes are altered most frequently. Pancreatic cancer cells harbor intact apoptotic machinery, but preferentially activate the autophagic pathway in response to triptolide [55]. The choice of the autophagic cell death pathway could depend on the metastatic potential of the cell lines being more metastatic than the others, which merits further investigation.

### 7.2. Signaling Pathways in Autophagy

Although many signaling pathways regulate autophagy, signaling from the cytoplasm to the autophagy machinery is mainly controlled in a negative manner through a serine/threonine kinase, mTOR. Akt, a positive regulator of mTOR, suppresses the formation of autophagosomes and inhibits autophagy [56]. The PI3K/Akt pathway can be deregulated via different mechanisms, as demonstrated for various cancers, including constitutive activation of growth factor receptors, PI3K amplification/mutation, inactivation of PTEN, amplification of Akt, and mutational activation of Akt itself [57]. Interestingly, overexpression of Akt, and the inactivation and loss of PTEN are frequently observed in pancreatic cancers [58,59], indicating that the pathway is a putative autophagic target in pancreatic cancers. Triptolide-induced autophagy is associated with inactivation of the Akt/mTOR/p70S6K pathway and up-regulation of the ERK1/2 pathway [55]. In addition, the role of the Ras/Raf-1/ERK1/2 signaling pathway in autophagy was confirmed in the human colon cancer cell line HT-29 [60]. Although increasing evidence implicates the importance of autophagy in cancer and tumor development, the fundamental question, whether autophagy kills cancer cells or protects them from unfavorable conditions, remains controversial. The threshold between autophagy as a cytoprotective process or programmed cell death is difficult to establish and probably depends on the extent of degradation of cellular components. Pro-apoptotic protein Bid may serve as a molecular switch between apoptosis and autophagy. For example, Bid knock down in MCF-7 cells exposed to CPT leads to a shift of cell death from apoptosis to autophagy. Since pro-apoptotic genes undergo mutations in malignant cells, the ability of cancer cells commitment to autophagy may have important therapeutic implications.

### 7.3. ER Stress in Autophagy

The ER functions to synthesize proteins, allow proper folding of secretory and transmembrane proteins, and stores high intracellular calcium that can activate calcium-dependent chaperone proteins that assist in protein folding. Disruption of any of these processes, such as by glucose depletion, which prevents the proper glycosylation of proteins, or by alterations in calcium homeostasis, such that calcium-dependent chaperones cannot function properly, leads to the accumulation of misfolded proteins that triggers ER stress [61]. Often seen as a protective mechanism, this build-up of misfolded proteins and subsequent stress leads to the unfolded protein response (UPR) mediated by three ER-localized transmembrane proteins that act as sensors: protein kinase RNA-like ER kinase (PERK), inositol requiring-1α (IRE1α), and activating transcription factor-6 (ATF6) [62]. Recently, it has been shown that prolonged ER stress can also lead to cell death mediated by autophagy [63]; therefore, while typically believed to be protective, excessive ER stress can instead result in autophagic cell death. Pancreatic epithelial cells have a highly developed ER due to heavy engagement in insulin and digestive enzyme secretion, and it has been suggested that they may be particularly sensitive to ER stress-induced apoptosis. By targeting the ER, a proteasome inhibitor, bortezomib, may be an effective therapy for pancreatic cancer, which may be hypersensitive to protein aggregation and subsequent ER stress-mediated apoptosis [64]. Cannabinol induces human pancreatic cancer cell death through the stimulation of autophagy. Cannabinol induced ceramide accumulation and eIF2α (eukaryotic initiation factor 2α) phosphorylation and thereby activated an ER stress response that promoted autophagy via inhibition of the Akt/mTORC1 axis [65].

## 8. Concluding Remarks

Pancreatic cancer remains one of the most lethal malignancies, and patients with metastatic pancreatic cancer have a bleak prognosis. The high mortality can be attributed to late diagnosis, rapid disease progression, and poor response to chemotherapy or radiotherapy. Traditional cancer therapy evokes cell death by inducing apoptosis; however, the apoptotic resistance inherent in cancer cells has been a significant barrier to effective chemotherapy. Autophagy is defined as a survival mechanism during stress conditions and a cell death pathway when apoptotic cell death mechanisms are deficient. As a result of the survival effects of autophagy, which were initially observed *in vitro*, the suggestion is that autophagy inhibition, in combination with standard chemotherapy, would be beneficial for tumor therapy. Better understanding of the genetic and epigenetic alterations, cellular and molecular signaling mechanisms, and apoptotic and autophagic mechanisms of pancreatic cancer would offer opportunities to develop novel therapeutics. HDAC inhibitors can induce both mitochondria-mediated apoptosis and caspase-independent autophagic cell death. Induction of autophagic cell death by HDAC inhibitors has clear clinical implications in treating cancers with apoptotic defects.
